# The Inter-Rater and Intra-Rater Reliability of the Actual Aquatic Skills Test (AAST) for Assessing Young Children’s Motor Competence in the Water

**DOI:** 10.3390/ijerph19010446

**Published:** 2021-12-31

**Authors:** Lisa Mertens, Kristine De Martelaer, Arja Sääkslahti, Eva D’Hondt

**Affiliations:** 1 Research Unit on Movement and Nutrition for Health and Performance, Department of Movement and Sport Sciences, Faculty of Physical Education and Physiotherapy, Vrije Universiteit Brussel, Pleinlaan 2, 1050 Brussels, Belgium; kdmartel@vub.be (K.D.M.); eva.dhondt@vub.be (E.D.); 2 Faculty of Sport and Health Sciences, University of Jyväskylä, P.O. Box 35 (L), FI-40014 Jyväskylä, Finland; arja.saakslahti@jyu.fi

**Keywords:** water competence, swimming, aquatic literacy, physical education, teacher, coach, assessment, pictorial scale

## Abstract

As children’s actual aquatic skills are important for the prevention of drowning as well as their engagement in lifelong aquatic physical activity, researchers and practitioners should be able to assess this vital concept accurately and reliably. Therefore, this study aimed to investigate the inter-rater and intra-rater reliability of the Actual Aquatic Skills Test (AAST), consisting of 17 different test items for the assessment of young children’s motor competence in the water. Six raters received a training and evaluation session on scoring the AAST, after which five of them assessed four test videos (of various children (*n* = 38) performing the test items) twice, with one to two weeks in between (i.e., test and re-test). Inter-rater and intra-rater reliability were determined per test video and for the different AAST test items across videos using Gwet’s Agreement Coefficient 2 (Gwet’s AC2). The Gwet’s AC2 for inter-rater reliability at the test varied from 0.414 to 1.000, indicating a moderate to perfect agreement between raters. For intra-rater reliability, it ranged from 0.628 to 1.000, demonstrating a good to perfect agreement between test and re-test scoring. In conclusion, the AAST is a promising tool to reliably assess young children’s actual aquatic skills in an indoor swimming pool.

## 1. Introduction

The concept of ‘water competence’ was first introduced and described by Langendorfer & Bruya (1995), focusing on fundamental motor skills in the water serving as the basis for further aquatic development [1]. Subsequently, Moran (2013) considered the concept of water competence from a drowning prevention perspective, defining it as “the sum of all personal aquatic movements that help prevent drowning, as well as the associated water safety knowledge, attitudes and behavior that facilitate safety in, on and around the water” [2]. More recently, Stallman et al. (2017) made the definition of the term water competence more tangible through their evidence-based proposal of 15 specific aquatic competencies, including physical, cognitive, as well as affective competencies [3]. In this paper, our focus will specifically be on the physical aspect within the concept of water competence, hereafter referred to as children’s actual aquatic skills.

Drowning is the world’s third leading cause of injury-related death, claiming more than 320,000 lives per year [4,5]. As to fatal and non-fatal drownings, young children are the most vulnerable age group, with one to four-year-olds and five to nine-year-olds experiencing the highest and second highest risk of drowning, respectively [4,5]. The World Health Organization (WHO) (2017 and 2020) highlights water safety education as one of the key approaches to prevent drowning amongst children [4,6]. The value of aquatic education as a preventive measure against drowning has also been supported by two-large scale retrospective studies conducted in different continents, comparing the aquatic educational backgrounds of young children who were victims of fatal drownings against that of a demographically equivalent control group [7,8]. Although these studies did not investigate or report the content of swimming lessons as well as the protective value of specific aquatic skills, they both indicate that aquatic education has the ability to reduce young children’s risk of drowning [7,8].

Numerous studies concerning land based motor skill performances already demonstrated a clear positive relationship between children’s level of motor competence and both their health-related physical fitness and physical activity levels [9,10,11,12]. This beneficial association between developing foundational skills and physical activity across the lifespan might also be valid in an aquatic environment, because an adequate aquatic skill level would enable children to engage in a broad array of aquatic activities and water-based recreation and sports, both now and in the longer term [13,14,15,16,17,18]. As children’s actual level of aquatic skills may not only be important for their water safety but also for their general health and well-being through engagement in aquatic oriented physical activity, it is crucial that both researchers and practitioners (e.g., swimming instructors and physical education teachers) are able to assess this vital concept accurately and reliably.

In addition, swimming instructors and physical education teachers should be able to clearly communicate young children’s actual level of aquatic skills and its importance to the child(ren)’s parents or primary caregivers, since this awareness might influence the degree of supervision deemed necessary when their child is being active in, on or around water [19]. A lack of supervision is a known risk factor for drowning [20]. Being correctly informed and aware of their child(ren)’s actual aquatic skill level, parents can correspondingly stimulate and support their child(ren)’s engagement in aquatic activities in view of pursuing a physically active lifestyle, considering that parental support is an important factor for children’s physical activity engagement [21,22]. Finally, lifeguards must also have the ability to correctly estimate or determine children’s actual aquatic skill levels in view of guaranteeing their water safety.

A promising new tool for assessing young children’s actual aquatic skill level that has yet to be proven reliable and valid, is the Actual Aquatic Skills Test (AAST), first administered by D’Hondt et al. (2021) [23]. The AAST [23] was based on transferring the 17 aquatic skills or test items included in the Pictorial Scale for Perceived Water Competence (PSPWC) [24] to an actual performance by the child in the water of an indoor swimming pool. Each of these 17 aquatic skills has to be assessed based on three possible levels of execution. For each level, a pictogram and corresponding performance criteria have been described in the PSPWC manual of Morgado et al. (2020) [24]. The derived AAST [23] is a tool worth investigating for a number of reasons, as elaborated below.

First, to the best of our knowledge and also according to other authors [23,25,26], there are currently only a few validated instruments available for assessing (pre)school children’s actual aquatic skills, which is in sharp contrast to the wide variety of assessment tools available for evaluating their actual level of motor competence on land [25,27,28,29]. To date, the majority of research investigating water competence in children, and more specifically their aquatic skills, is based on self-reported or parent proxy-reported estimates thereof [3,30,31,32]. This approach is generally found to be less labor-intensive and time-consuming than actually assessing children’s aquatic skill level in the water [30,33,34]. Nonetheless, it is known that both children’s self-perception as well as the parental perception of children’s level of (aquatic) skills are not always in accordance with reality [23,35,36,37]. Estevan et al. (2017) [36], for example, only found weak to moderate positive relationships between children’s actual skill level (on land) and their self-perception as well as the parental perception and the perception of physical education teachers, with the children themselves being the most inaccurate when estimating their own skill level. Additionally, in practice, the evaluation of aquatic skills by teachers or coaches often refers to their experience, if not their intuition [29].

Second, the main and often sole determinant of children’s actual aquatic skill level considered in previous studies, but also by most swimming instructors, lifeguards and parents or caregivers, is the ability of a child to swim a certain distance [38]. Although propulsion in the water is a fundamental aquatic skill, the evaluation of propulsion alone is inadequate in terms of assessing a child’s water safety [3,30,38]. Mastering propulsion does not guarantee that the child is able to breathe, rest, call for help or climb out of the water [3,39,40]. Furthermore, Stallman (2008) [41] identified the underestimation of possible danger and unexpected occurrences, such as cold water, a strong current or falling from great height, as common causes of drowning.

Third, the content of the AAST [23] aligns perfectly with that of the PSPWC [24], allowing research such as that of D’Hondt and colleagues [23], who examined the relationship between the actual and the self- and parent-perceived aquatic skill level of children. According to Stallman et al. (2017) [3], a vital element of water competence is the individual’s ability to accurately estimate their own actual aquatic skill level. More specifically, one’s self-perceived aquatic skill level has to correspond closely to one’s actual skill level in order to appropriately judge potential risks and constraints when being in, on and around water. As such, children who overestimate their actual aquatic skill level may have a higher chance of drowning [3,42,43,44,45]. Moreover, children who underestimate their actual aquatic skill level might avoid water and participation in aquatic activities, therefore losing out on chances to be physically active in this particular setting or movement environment. When swimming instructors and physical education teachers are able to map an individual child’s actual and perceived aquatic skill profile, they can counter these potential threats and/or inform the parents or caregivers about them. In order to investigate the relationship between one’s actual and perceived level of aquatic skills, an alignment between test instruments measuring both constructs is recommended [46,47]. However, only a few of the scarce studies examining and comparing children’s actual and perceived motor competence in the water used aligned instruments, since there are currently few (aligned) test instruments available in this regard [23,37,48].

Furthermore, D’Hondt et al. (2021) already demonstrated a good internal consistency of the 17 test items included in both the PSPWC [24] and the AAST [23]. Besides, given that the AAST [23] has been derived from a pictorial scale questionnaire developed for children, the evaluation of the different test items is fairly uncomplicated. Additionally, the pictograms included in the PSPWC [24] may facilitate an easy understanding and administration of the AAST [23] in areas with low literacy levels.

Consequently, the AAST [23] can be considered a promising tool for assessing the motor competence of young children in the water. Therefore, the purpose of this study is to investigate both the inter-rater and intra-rater reliability of the AAST [23] for the assessment of young children’s actual aquatic skill level (using video footage).

## 2. Materials and Methods

Our research approach was approved by the local Medical Ethics Committee at VUB (B.U.N. 143201942643) on 16 September 2020.

### 2.1. Raters

In total, six participant raters (i.e., one female and five males, aged 21–24 years) were conveniently and voluntarily recruited from students enrolled in the Master in Movement and Sport Sciences program of the Vrije Universiteit Brussel (VUB, Belgium) to provide AAST [23] evaluations. Study participants were excluded if they were not able to attend an organized training session for raters. As a part of obtaining their Bachelor’s degree in Physical Education and Movement Sciences, all of the recruited participant raters (*n* = 6) followed weekly one-hour swimming classes themselves for a total of five consecutive semesters, with each semester consisting of 13 class weeks. In addition, all of the participant raters, with the exception of rater F, had experience teaching at least 10 swimming classes. Furthermore, one of them was a certified physical education teacher (i.e., rater B). Another participating rater completed a specialized water safety course, achieving his lifeguard license (i.e., rater E). Lastly, a third rater was a former competitive swimmer, and was still an active swimming coach during the conduct of this reliability study (i.e., rater D).

### 2.2. Subjects

Due to COVID-19 and the subsequent closure of public swimming pools during the envisaged data collection period, live administration of the Actual Aquatic Skills Test (AAST) [23] with children in the water of an indoor swimming pool was not possible. Therefore, the raters were asked to assess previously obtained video recordings of different children performing the 17 aquatic skill test items included in the AAST [23]. These recordings were grouped into multiple composite videos (*n* = 8), using the Shotcut video editing application [49], including three composite videos for the purpose of training the raters and familiarizing them with the testing protocol (i.e., training videos), one composite video to evaluate the accuracy of the raters against experts (i.e., evaluation video) and four composite videos for determining the inter-rater and intra-rater reliability of the AAST (i.e., test video 1, test video 2, test video 3 and test video 4).

### 2.3. Procedure

The video recordings, employed for this investigation, were originally obtained for the purpose of a previous study of our research group [23], for which 134 children between six and nine years of age were recruited through convenience sampling, in collaboration with multiple swimming schools located in Flanders and the Brussels Capital Region (Belgium), by either addressing their parent(s) or guardian(s) personally at the swimming pool or via e-mail. These swimming schools were intentionally selected because of their focus on developing children’s fundamental and survival aquatic skills before teaching them competitive swimming strokes. The live administration and video recording of the AAST [23] performed by these children took place under the supervision of one of the main researchers who was also an experienced swimming teacher. Subsequently, all collected video recordings were carefully screened by the lead author of this reliability study. Bad quality recordings and recordings in which the child gave the impression not to understand the AAST task(s) [23], were excluded in view of composing the training, evaluation and test videos.

Each composite video contained the test instructions and 17 included recordings (i.e., one for every test item of the AAST [23]), applying the same order of test items as presented in the PSPWC manual [24]. Every test item within the evaluation video and each composite test video was performed by a different child (i.e., 17 different children per composite video or set of recordings). In total, for the four composite test videos, video recordings of the performances of 38 different children were used. A white screen (lasting for 4.0 s) always separated the performance of two subsequent tasks or test items. This was the only moment where the raters were allowed to pause the video, in order (1) to be able to (re)check the task criteria in the PSPWC manual [24], (2) to fill in the provided score-sheet (Table 1), and (3) to prepare themselves for assessing the next test item performed by a different child. Under no circumstances were raters allowed to fast-forward or rewind any parts of the video. Every recorded task performance of each child per test item was shown twice in the composite video with these identical performances being divided by a black screen (lasting for 1.5 s). The scores to be awarded and the associated scoring criteria were exactly the same as described in the PSPWC manual [24]. That is, ‘1’—the child is not able to perform the aquatic skill, ‘2’—the child is partly able to perform the aquatic skill and thus still in progress, and ‘3’—the child is able to correctly perform the aquatic skill. Table 2 provides an overview of the three different mastery levels for each of the 17 aquatic skills of the PSPWC [24]. In the PSPWC manual [24], the minimum criteria for each mastery level of every aquatic skill or test item are described in more detail (see Table 3). The AAST assessment used in this reliability study was based upon these minimum criteria. In order to achieve a score of ‘3’ on a certain aquatic skill or test item, the child’s performance had to meet all the criteria described for level 3 of that particular test item. Consequently, when one or more criteria were not fulfilled, a score of ‘2’ was granted, provided that all the criteria for level 2 were met. When this was not the case, the child’s performance was to be awarded a score of ‘1’.

### 2.4. Training

Prior to the actual video assessments in the context of this reliability study, all participating raters received a two-hour online training session (in separate groups of three), provided by the lead investigator. During this online training session, the PSPWC [24] and the AAST [23] testing protocols were explained in detail. Consequently, the raters independently assessed a total of three different composite videos (i.e., training videos), each of which contained the 17 PSPWC/AAST test items [23,24] performed by different children in the water of an indoor swimming pool. The raters were urged to read the minimum criteria per score level for each aquatic skill task in the PSWPC manual [24] both before and after watching a child’s performance on a specific test item to be assessed. After individually scoring the 17 performances within one composite training video, the differences in scoring between the lead investigator and the raters as well as between the raters themselves were discussed after each video assessment until consensus was reached. When the scoring criteria were not applied correctly (e.g., a rater assigning a score of 3 to a child’s performance for a particular aquatic skill and at the same time stating that a certain criterion for level 3 of that test item was not met), the correct assessment method was explained again.

### 2.5. Evaluation

One week after the online training session, the participating raters received the so- called evaluation video. In order to further progress and participate in this reliability study as a rater, participants had to establish an 80% agreement with the scores awarded by international experts in the field, which is in accordance with the criterion used in similar reliability studies investigating land based motor skill assessment tools [50,51]. These experts were four co-developers of the PSPWC [24], who also assessed the same evaluation video or set of recorded test items performed by 17 different children. These four international experts awarded the very same score to 11 out of 17 child performances on the AAST [23] (i.e., test items 1, 2, 3, 4, 5, 7, 9, 11, 12, 13 and 14). Regarding four other aquatic skill performances (i.e., test items 6, 8, 15 and 17) their opinions were divided, with three experts opposed to one. In this case, the score awarded by the majority of the experts was used as the ‘gold standard’. When the opinions were split, two experts opposed to the other two (i.e., test items 10 and 16), there was a group discussion until consensus was reached or until the majority agreed on the final score to be awarded. All six participant raters, who followed the online training session for AAST [23] assessment (i.e., rater A, B, C, D, E and F), achieved an accuracy above 80% against the experts regarding the scoring of the evaluation video (i.e., 94% for rater A and C, 88% for rater B, C and D, and 82% for rater F). The only female rater (i.e., rater F) dropped out with regard to the next study phase due to personal matters. Therefore, one week after successfully completing the evaluation video, five remaining raters individually assessed the four test videos within a timeframe of one week after receiving the video footage (i.e., test). After another week in which the raters were not allowed to look at or score any of the videos, these raters were asked to score the same four test videos a second time, again being provided with a one-week timeframe to complete this task (i.e., re-test). This minimum test-retest interval of one week is a commonly used in reliability studies of motor skill assessment tools [52].

### 2.6. Statistical Analysis

Both inter-rater and intra-rater reliability were determined for each of the four test videos and separately for each individual test item across the four composite videos. Inter-rater reliability was solely determined for the test, and thus not for the re-test. To investigate both the inter-rater and intra-rater reliability of the ordinally scored AAST [23] data, the Gwet’s Agreement Coefficient 2 (Gwet’s AC2) [53] was determined by means of the Real Statistics Resource Pack for Microsoft Excel [54]. The use of Gwet’s AC2 was a deliberate decision, given its resistance to an unbalanced distribution of data across the scoring categories in contrast to other reliability measures [55,56]. Gwet’s AC2 values can range from 0 to 1, where 0 represents absolute disagreement and 1 represents perfect agreement. In accordance with the classification used by Thoenen et al. (2021) [57], Gwet’s AC2 values below 0.20 were considered to represent a poor level of agreement, whereas Gwet’s AC2 values ranging between 0.21–0.40, 0.41–0.60, 61–0.80 and 0.80–1.00 were considered to demonstrate a fair, moderate, good and very good level of agreement, respectively. Additionally, for inter-rater reliability, the scoring distribution for each test item per test video was reported and expressed as the percentage of raters per awarded score presented in an agreement table. For intra-rater reliability, the percent agreement between test and re-test scoring was also reported for each rater (i.e., the percentage of test items that were awarded the exact same score for the test and the re-test on an individual rater basis).

## 3. Results

### 3.1. Inter-Rater Reliability

As demonstrated in Table 4, Gwet’s AC2 values for inter-rater reliability of the test videos varied between 0.646 and 0.922, demonstrating a good level of agreement for test videos 1, 2 and 3, whereas test video 4 showed a very good level of agreement between the different raters.

Regarding the inter-rater reliability per test item across the four test videos, Gwet’s AC2 values ranged from 0.414 (i.e., moderate agreement) to 1.000 (i.e., perfect agreement). Test items 1, 2, 4 and 9 demonstrated perfect agreement among all raters. A very good level of agreement was found for test items 8, 11, 15 and 17, whereas test items 5, 7, 10, 12, 13, 14 and 16 showed good inter-rater reliability. A moderate agreement was found for test items 3 and 6. The scoring distribution for each aquatic skill task per test video, expressed as the percentage of raters per awarded score, is presented in Table 5.

### 3.2. Intra-Rater Reliability

The Gwet’s AC2 values and the percent agreements for the intra-rater reliability are presented in Table 6 and Table 7, respectively. The intra-rater reliability across the four test videos was found to be very good for all raters, with rater B obtaining the highest level of agreement, followed by rater A, C, D and E, respectively. The Gwet’s AC2 values for the intra-rater reliability per test video varied between 0.834 (i.e., very good agreement) and 1.000 (i.e., perfect agreement). The intra-rater reliability per test item ranged from 0.628 (i.e., good agreement) to 1.000 (i.e., perfect agreement). All raters achieved perfect intra-rater reliability for test items 2, 4, 11, 14, 15 and 17. For test items 1, 6, 7, 8, 12 and 16, four out of the five raters achieved perfect agreement, whereas the remaining rater always achieved a good level of agreement between test and re-test scoring. For test items 3, 5, 9, 10 and 13, three out of the five raters obtained a Gwet’s AC2 of 1.000, indicating a perfect agreement between test and re-test scoring, whereas the remaining two raters always obtained a good level of agreement in terms of their intra-rater reliability. In other words, rater A and rater B demonstrated perfect agreement between test and re-test scoring for all but two test items of the AAST [23]. Rater C and D obtained perfect intra-rater reliability for 14 out of the 17 aquatic skills, whereas rater E obtained perfect intra-rater-reliability for 11 out of the 17 AAST items [23].

## 4. Discussion

The purpose of this study was to assess both the inter-rater and intra-rater reliability of the AAST [23], a test instrument for assessing young children’s actual aquatic skill level based on transferring the 17 test items from the PSPWC [24] to an actual performance of those aquatic skill tasks in the water of an indoor swimming pool.

Regarding inter-rater reliability, all composite test videos demonstrated a good to very good agreement. When looking at the inter-rater reliability of the individual test items across videos, four out of the 17 test items (i.e., test items 1,2, 4 and 9) demonstrated perfect agreement, another four test items demonstrated a very good level of agreement (i.e., test items 8, 11, 15 and 17) and six test items (i.e., test items 5, 7, 10, 12, 13,14 and 16) demonstrated a good level of agreement. It must be noted that all raters awarded the very same score (i.e., score 3) to all child performances of test item 2 (i.e., standing and submersion in the water), indicating that there was little to no variation in children’s level of skill performance for this particular aquatic skill across the test videos to be scored. The lowest level of agreement (i.e., moderate agreement) was found for test items 3 and 6. The moderate agreement for test item 3 (i.e., blowing bubbles under water) resulted predominantly from the stricter assessment of one rater (i.e., rater E) when compared to the other four raters. Rater E awarded a lower score to the performance of test item 3 in test videos 1 and 2. For test video 3, both rater A and rater E awarded a lower score to the child’s performance when compared to the other three raters. This degree of disagreement between the raters regarding test item 3 was likely caused by the use of two-dimensional video recordings taken from the edge of the swimming pool, making it more difficult to determine whether the child was actually blowing bubbles under water or whether the bubbles were a result of the child moving in the water. This is in accordance with the findings of Vogt & Straub (2020), who also found a lower inter-rater reliability for the video recorded task of blowing bubbles under water, compared to other basic aquatic skills [29]. Another possible explanation is that the short duration for which the children performed the aquatic skill (with two out of four children blowing bubbles for less than 1.5 s), made the assessment more challenging. Adding an extra criterion to the PSPWC [24] regarding the minimal duration a child has to blow bubbles might enhance the reliability of scoring this item. Despite the finding of a moderate level of agreement, test item 6 (i.e., front star) showed the lowest inter-rater reliability. Two of the four children performing this particular test item only briefly maintained the front star or were moving while floating. Therefore, the inclusion of additional criteria in the PSPWC test manual [24] regarding a minimal duration and also regarding the amount of movement that is (not) allowed while a child is performing the front star might benefit the agreement between raters. Regarding intra-rater reliability, the level of agreement between test and re-test scoring per test video was very good to perfect. Considering the intra-rater reliability per test item across all four test videos, the level of agreement was either perfect or very good. A perfect level of agreement between test and re-test scoring was obtained by all the raters for six test items (i.e., test items 2, 4, 11, 14, 15 and 17), by four out of the five raters for five test items (i.e., test items 1, 6, 7, 8, 12 and 16) and by three out of the five raters regarding the remaining five test items (i.e., test items 3, 5, 9, 10 and 13). However, due to the limited amount of child performances per test item (*n* = 4), it was not possible to obtain a very good level of agreement for a specific test item. Once a participant rater awarded one of the four child performances on a particular test item a different score during the re-test than during the test, the intra-rater reliability always dropped from a perfect to a good level of agreement. Therefore, future reliability research regarding the AAST [23] should pursue a greater amount of child performances to be assessed for each of its test items.

D’Hondt et al. (2021) [23] already demonstrated a good internal consistency of the 17 test items included in both the PSPWC [24] and the AAST [23], indicating that these tools measure one overall construct (i.e., perceived vs. actual water motor competence in children, respectively). In combination with the findings of the present study, indicating that the AAST [23] also is a reliable assessment tool, it can be said that the AAST [23] has the ability to meet the need for a sound test instrument to assess young children’s actual aquatic skills, being appropriate for use by both researchers and practitioners.

In the present study, previously obtained video recordings of children performing the 17 different test items of the AAST [23] in the water of an indoor swimming pool were used. Although the AAST [23] does not require the video recording of these performances, since live administration is the usual way of working, the video recordings composed with the Shotcut video editing application [49] enforced the participating raters to watch the same performance of a child for a second time. Furthermore, the use of video recordings made it possible to include the performances of 17 different children within each composite test video, with each child performing only one of the 17 test items of the AAST [23] to ensure sufficient variation in aquatic skill level within one overall AAST test video. As such, the participant raters were stimulated to look at each aquatic skill task performance with a fresh pair of eyes, not being biased by the child’s performance on one of the previous test items. Besides, the composition of video recordings of different children reduced the chance of the raters recalling any of the children and their performances [58]. As shown in the present study, video recordings might be especially useful for training purposes, allowing both researchers and practitioners to become familiar with the PSPWC-based AAST test protocol and its scoring [23,24]. Likewise, video recordings have also been used effectively in previous studies investigating children’s motor competence assessment on land [51]. Finally, video recordings can also be used as an educational tool for teaching children and their aquatic skill assessors as provided in the context of blended learning [59].

### 4.1. Strengths and Weaknesses of the Study

Our study demonstrated several strengths. First of all, the reliability research conducted contributes to the limited literature regarding test instruments for assessing young children’s actual aquatic skill level, not relying on self-reports or parent-proxy reports. Second, the included skills in the AAST [23] and PSPWC [24] cover more than solely a child’s swimming ability, as they were carefully selected for their importance as fundamentals for further aquatic skill development and in regard to water safety, which is especially important for young children considering they have the highest chance of drowning compared to other age groups [3,4,5]. Third, the AAST [23], which serves as an instrument for assessing young children’s actual aquatic skill level (i.e., the physical aspect of water competence), perfectly aligns with the PSPWC [24] for assessing their perceived aquatic skill level, allowing (1) future research to further examine the important and possibly developmental variable relationship between these two vital concepts, and (2) practitioners and parents or caregivers to compare children’s actual and perceived aquatic skill level to better estimate and respond to the child’s individual needs [3]. Finally, the raters participating in this study were all students enrolled in the Master in Movement and Sport Sciences, having experience with aquatic education and movement analysis being or potentially becoming practitioners (e.g., physical education teachers, swimming instructors and lifeguards) for whom it is important to know how to assess and thus estimate young children’s actual aquatic skill level correctly.

On the other hand, this reliability study also has some limitations to be considered. A downside of using video footage is that the raters were not able to adjust their point of view and had to settle for the pool edge camera’s perspective of the child(ren)’s performance in the water. Furthermore, the video recordings only allowed for a two-dimensional perception of the performances of the different test items. As highlighted above, the lack of depth perception might have had a negative influence on the assessment of test item 3 (i.e., blowing bubbles under water), for example. The use of video footage allowed the raters to read the task criteria both before and after watching a child’s performance, which is rather unrealistic during live administration with large(r) groups of children and a limited amount of time, such as in a school or training context. Moreover, in this study the determination of the intra-rater reliability lacked sensitivity due to the limited amount of children (*n* = 4) performing a specific test item.

### 4.2. Future Directions and Practical Implications

Future research should thus further investigate the reliability of the AAST [23], when used to assess young children’s aquatic skills during live administration in the water of an indoor swimming pool [51]. Moreover, in order to examine the inter-rater and intra-rater reliability of the individual test items of the AAST (*n* = 17), the inclusion of a greater amount of individual child performances for each test item as well as a greater variation in mastery levels of aquatic skill between those performances than in the present study is advised. Investigating the AAST’s [23] reliability between and within raters who are active physical education teachers and swimming instructors is a direction for future research. Another avenue for future research is to explore the relationship and possible (mis)match between children’s actual AAST (measured by physical education teachers and/or swimming instructors) and the “intuition” of parents and lifeguards. Furthermore, the administration of the AAST [23] in other aquatic environments also seems a relevant avenue for future research, as drownings frequently occur in open water [20]. A long term vision on research regarding the AAST includes investigating its predictive validity in terms of drowning incidences. From a research perspective, the AAST [23] also presents itself as a useful tool for examining the correlates of young children’s actual aquatic skill levels, for determining and (internationally) comparing young childhood populations’ actual aquatic skill level as well as for evaluating intervention programs targeting to improve young children’s actual aquatic skill level. The importance of sharing data internationally is stressed by the United Nations General Assembly (2021), as it is critical for the successful global implementation of children’s water safety interventions and programs [60].

From a practitioner’s point of view, the AAST [23] is applicable for evaluation purposes and tracking the progress of a child’s actual aquatic skill level. Moreover, the instrument lends itself to assessment for learning progress (i.e., stimulating the learning process through assessment [61]) and for planning and/or individually tailoring the lessons with the appropriate lesson content (i.e., skill levels or aquatic activities). Using the AAST [23], the physical education teacher and the swimming instructor can also (better) inform parents and caregivers about their child(ren)’s actual aquatic skill level and its significance in terms of water safety and future participation in aquatic oriented activities [62]. Additionally, the PSPWC [24] as a pictorial scale can be administered by the practitioners but also the parents themselves. In combination with the results of a child’s performance on the AAST [23] in the water, this empowers practitioners and parents to sufficiently supervise, support and stimulate their child(ren) regarding their engagement in aquatic activities. Administering the AAST [23] among a large group of students might seem impractical and time-consuming, however, not every skill of every child has to be assessed in one single lesson. As stated earlier, the AAST [23] can be used to plan and tailor aquatic education lessons. In that case, each lesson might specifically focus on a particular subset of skills (e.g., the rotations), which can then be assessed. The AAST [23] might also be used as a continuous progress tracker over a period of time. When a child shows their ability to perform a particular aquatic skill from the AAST [23] at a certain level during a lesson or even during free play, this can simply be noted and registered to keep track of the individual progress being made in terms of the physical aspect of water competence.

## 5. Conclusions

Regarding the inter-rater reliability of the AAST [23], this study found a good to very good level of agreement between raters for the four AAST test videos included. For the inter-rater reliability of the individual AAST test items [23] across these test videos, a moderate to perfect level of agreement was found, with test item 3 and test item 6 being the only test items demonstrating a moderate level of agreement. Considering the intra-rater reliability of the AAST [23], the level of agreement between test and re-test scoring for the four AAST test videos ranged from very good to perfect for all the raters involved. For the individual AAST test items [23], the level of agreement between test-and retest scoring was considered to be good to perfect. In conclusion, this study demonstrated that the AAST [23] is a useful and reliable tool for assessing and monitoring young children’s actual aquatic skill levels. A translation of this reliability study to a live administration and scoring of the AAST [23] in the context of a swimming pool is required to confirm these promising results and to move both research and practice within this specific field forward.

## Figures and Tables

**Table 1:** Rater’s score sheet example.

What Is the Appropriate Level for the Child Observed Per Test Item?		
‘1’ = not able to perform the aquatic skill		
‘2’ = partly able to perform the aquatic skill		
‘3’ = able to correctly perform the aquatic skill		
Aquatic skill or	Description	Score:
test item		‘1’, ‘2’ or ‘3’.
1	Lying down in a prone position using hands on the bottom to move forward (as a crocodile)	
2	Standing and submersion in the water	
3	Blowing bubbles under water	
4	Catching an object under water	
5	Floating on the back (i.e., back star)	
6	Floating on the front (i.e., front star)	
7	Water entry by slide	
8	Pushing from the wall and gliding under water	
9	Leg propulsion on the back	
10	Leg propulsion on the front	
11	Water entry by jumping	
12	Water entry by diving	
13	Water exit by climbing out	
14	Vertically treading water	
15	Turning from the front to the back in an aligned position	
	(i.e., longitudinal axis rotation)	
16	Changing direction while swimming on the front	
	(i.e., transverse axis rotation)	
17	Turning from the back to the front (i.e., sagittal axis rotation)	

**Table 2:** Overview of the three different mastery levels per test item as included in the Pictorial Scale of Perceived Water Competence (PSPWC) [24].

Level Aquatic Skill or Test Item	Unable to Execute the Aquatic Skill ‘1’	Partly Able to Execute the Aquatic Skill (in Progress) ‘2’	Fully Able to Execute the Aquatic Skill ‘3’
**Test item 1** Move forward as a crocodile	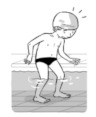	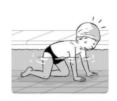	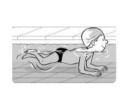
**Test item 2** Standing and submersion in the water	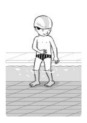	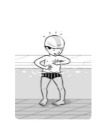	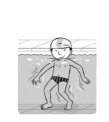
**Test item 3** Blowing bubbles under water	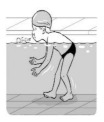	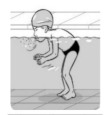	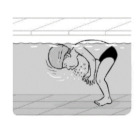
**Test item 4** Catching an object under water	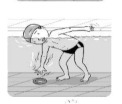	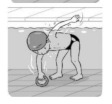	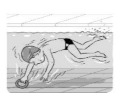
**Test item 5** Floating on the back (i.e., back star)	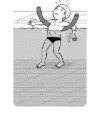	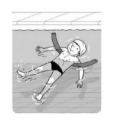	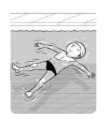
**Test item 6** Floating on the front (i.e., front star)	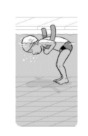	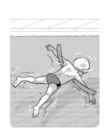	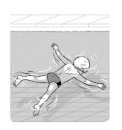
**Test item 7** Water entry by slide	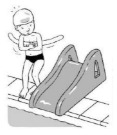	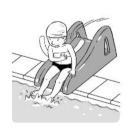	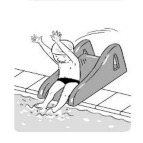
**Test item 8** Pushing from the wall and gliding under water	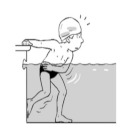	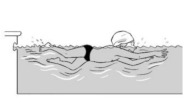	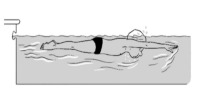
**Test item 9** Leg propulsion on the back	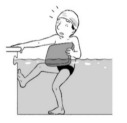	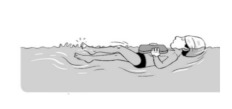	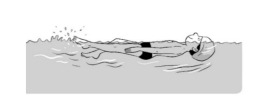
**Test item 10** Leg propulsion on the front	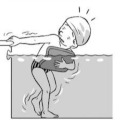	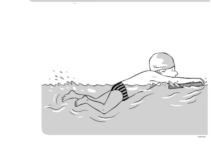	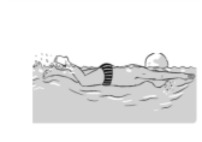
**Test item 11** Water entry by jumping	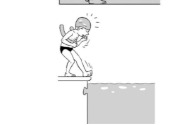	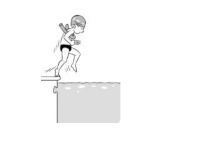	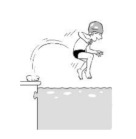
**Test item 12** Water entry by diving	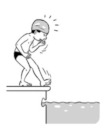	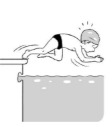	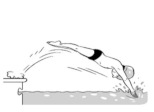
**Test item 13** Water exit by climbing out	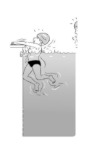	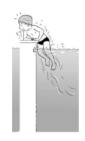	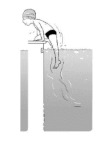
**Test item 14** Vertically treading water	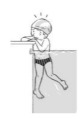	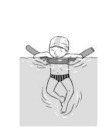	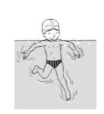
**Test item 15** Longitudinal axis rotation	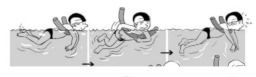	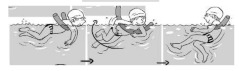	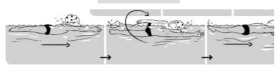
**Test item 16** Transverse axis rotation	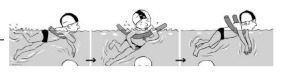	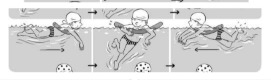	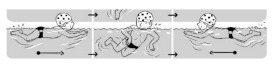
**Test item 17** Sagittal axis rotation	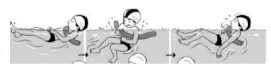	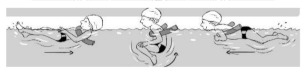	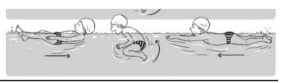

Permission to publish these pictures was granted by the developers of the PSPWC [24]. Permission to publish this table was granted by D’Hondt et al. (2021) [23].

**Table 3:** Example of the minimum criteria per mastery level for test item 1 of the PSPWC [24], when performed by the child in the water of an indoor swimming pool during the Actual Aquatic Skills Test (AAST) [23].

Test Item 1		
Lying down in a prone position using hands on the bottom to move forward		
(as a crocodile)		
Level		Minimum criteria
1	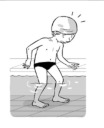	The child stands up in the shallow water but does not dare to lie down.
2	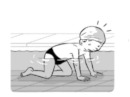	The child moves forward on all fours but is not completely submerged in the water.
3	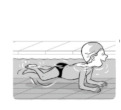	The child lies down in a prone position with arms or hands in contact with the bottom of the wading (paddling) pool, with the body extended and immersion up to the shoulders.

Permission to publish these pictures and criteria was granted by the developers of the PSPWC [24].

**Table 4:** Gwet’s AC2 test results for inter-rater reliability.

Test Component	AC2	LoA	SE Subj	CI 95% Subj	SE Tot	CI 95% Tot
Test video 1	0.794	G	0.083	[0.617; 0.971]	0.093	[0.596; 0.993]
Test video 2	0.646	G	0.091	[0.452; 0.839]	0.126	[0.379; 0.912]
Test video 3	0.701	G	0.104	[0.480; 0.921]	0.123	[0.440; 0.962]
Test video 4	0.922	VG	0.055	[0.806; 1.000]	0.062	[0.792; 1.000]
**Test item 1** Move forward as a crocodile	1.000	P	0.000	[1.000; 1.000]	0.000	[1.000; 1.000]
**Test item 2** Standing and submersion in the water	1.000	P	0.000	[1.000; 1.000]	0.000	[1.000; 1.000]
**Test item 3** Blowing bubbles under water	0.507	M	0.199	[−0.127; 1.000]	0.382	[−0.708; 1.000]
**Test item 4** Catching an object under water	1.000	P	0.000	[1.000; 1.000]	0.000	[1.000; 1.000]
**Test item 5** Floating on the back (i.e., back star)	0.671	G	0.194	[0.052; 1.000]	0.262	[−0.164; 1.000]
**Test item 6** Floating on the front (i.e., front star)	0.414	M	0.207	[−0.246; 1.000]	0.310	[−0.571; 1.000]
**Test item 7** Water entry by slide	0.713	G	0.194	[0.097; 1.000]	0.254	[−0.094; 1.000]
**Test item 8** Pushing from the wall and gliding under water	0.835	VG	0.179	[0.264; 1.000]	0.187	[0.240; 1.000]
**Test item 9** Leg propulsion on the back	1.000	P	0.000	[1.000; 1.000]	0.000	[1.000; 1.000]
**Test item 10** Leg propulsion on the front	0.634	G	0.235	[−0.114; 1.000]	0.273	[−0.234; 1.000]
**Test item 11** Water entry by jumping	0.863	VG	0.143	[0.408; 1.000]	0.199	[0.230; 1.000]
**Test item 12** Water entry by diving	0.777	G	0.219	[0.079; 1.000]	0.228	[0.051; 1.000]
**Test item 13** Water exit by climbing out	0.702	G	0.207	[0.043; 1.000]	0.237	[−0.053; 1.000]
**Test item 14** Vertically treading water	0.657	G	0.257	[−0.161; 1.000]	0.288	[−0.261; 1.000]
**Test item 15** Longitudinal axis rotation	0.806	VG	0.196	[0.181; 1.000]	0.204	[0.155; 1.000]
**Test item 16** Transverse axis rotation	0.780	G	0.138	[0.341; 1.000]	0.200	[0.143; 1.000]
**Test item 17** Sagittal axis rotation	0.828	VG	0.193	[0.215; 1.000]	0.196	[0.203;1.000]

AC2 = Gwet’s Agreement Coefficient 2; LoA = Level of Agreement; M = moderate (0.41–0.60); G = good (0.61–0.80); VG = very good (0.81–0.99); P = perfect (1.00); SE = standard error; CI = confidence interval; subj = subjects; tot = total.

**Table 5:** Agreement table for inter-rater reliability.

Test Item	Test Video	Percentage of Raters Per Awarded Score (%)		
		Score ‘1’	Score ‘2’	Score ‘3’
**Test item 1** Move forward as a crocodile	Test video 1	0	0	100
	Test video 2	0	100	0
	Test video 3	0	0	100
	Test video 4	0	0	100
**Test item 2** Standing and submersion in the water	Test video 1	0	0	100
	Test video 2	0	0	100
	Test video 3	0	0	100
	Test video 4	0	0	100
**Test item 3** Blowing bubbles under water	Test video 1	0	20	80
	Test video 2	0	20	80
	Test video 3	40	60	0
	Test video 4	0	100	0
**Test item 4** Catching an object under water	Test video 1	100	0	0
	Test video 2	0	0	100
	Test video 3	100	0	0
	Test video 4	0	0	100
**Test item 5** Floating on the back (i.e., back star)	Test video 1	0	40	60
	Test video 2	0	20	80
	Test video 3	0	0	100
	Test video 4	0	100	0
**Test item 6** Floating on the front (i.e., front star)	Test video 1	0	60	40
	Test video 2	60	40	0
	Test video 3	20	80	0
	Test video 4	0	0	100
**Test item 7** Water entry by slide	Test video 1	0	0	100
	Test video 2	0	20	80
	Test video 3	0	40	60
	Test video 4	0	0	100
**Test item 8** Pushing from the wall and gliding under water	Test video 1	0	0	100
	Test video 2	0	40	60
	Test video 3	0	0	100
	Test video 4	0	0	100
**Test item 9** Leg propulsion on the back	Test video 1	0	100	0
	Test video 2	100	0	0
	Test video 3	0	0	100
	Test video 4	0	100	0
**Test item 10** Leg propulsion on the front	Test video 1	0	100	0
	Test video 2	20	0	80
	Test video 3	60	40	0
	Test video 4	0	0	100
**Test item 11** Water entry by jumping	Test video 1	100	0	0
	Test video 2	0	0	100
	Test video 3	0	20	80
	Test video 4	100	0	0
**Test item 12** Water entry by diving	Test video 1	0	60	40
	Test video 2	100	0	0
	Test video 3	0	100	0
	Test video 4	0	0	100
**Test item 13** Water exit by climbing out	Test video 1	0	20	80
	Test video 2	0	60	40
	Test video 3	0	0	100
	Test video 4	0	0	100
**Test item 14** Vertically treading water	Test video 1	0	0	100
	Test video 2	0	0	100
	Test video 3	20	60	20
	Test video 4	0	20	80
**Test item 15** Longitudinal axis rotation	Test video 1	0	0	100
	Test video 2	0	40	60
	Test video 3	0	0	100
	Test video 4	0	100	0
**Test item 16** Transverse axis rotation	Test video 1	0	0	100
	Test video 2	0	20	80
	Test video 3	0	20	80
	Test video 4	0	0	100
**Test item 17** Sagittal axis rotation	Test video 1	0	0	100
	Test video 2	0	0	100
	Test video 3	0	0	100
	Test video 4	0	60	40

**Table 6:** Gwet’s AC2 test results for intra-rater reliability.

Test	Rater A				Rater B				Rater C				Rater D				Rater E			
Component	AC2	LoA	SE	CI 95%	AC2	LoA	SE	CI 95%	AC2	LoA	SE	CI 95%	AC2	LoA	SE	CI 95%	AC2	LoA	SE	CI 95%
Test video 1	0.848	VG	0.112	[0.610; 1.000]	1.000	P	0.000	[1.000; 1.000]	0.920	VG	0.081	[0.749; 1.000]	0.839	VG	0.114	[0.597; 1.000]	0.837	VG	0.112	[0.600; 1.000]
Test video 2	1.000	P	0.000	[1.000; 1.000]	1.000	P	0.000	[1.000; 1.000]	0.834	VG	0.116	[0.589; 1.000]	0.914	VG	0.086	[0.733; 1.000]	0.837	VG	0.112	[0.600; 1.000]
Test video 3	1.000	P	0.000	[1.000; 1.000]	1.000	P	0.000	[1.000; 1.000]	1.000	P	0.000	[1.000; 1.000]	1.000	P	0.000	[1.000; 1.000]	0.918	VG	0.082	[0.743; 1.000]
Test video 4	1.000	P	0.000	[1.000; 1.000]	0.855	VG	0.102	[0.637; 1.000]	1.000	P	0.000	[1.000; 1.000]	1.000	P	0.000	[1.000; 1.000]	0.919	VG	0.082	[0.744; 1.000]
**Test item 1** Move forward as a crocodile	1.000	P	0.000	[1.000; 1.000]	1.000	P	0.000	[1.000; 1.000]	1.000	P	0.000	[1.000; 1.000]	1.000	P	0.000	[1.000; 1.000]	0.660	G	0.357	[−0.477; 1.000]
**Test item 2** Standing and submersion	1.000	P	0.000	[1.000; 1.000]	1.000	P	0.000	[1.000; 1.000]	1.000	P	0.000	[1.000; 1.000]	1.000	P	0.000	[1.000; 1.000]	1.000	P	0.000	[1.000; 1.000]
**Test item 3** Blowing bubbles under water	1.000	P	0.000	[1.000; 1.000]	0.673	G	0.332	[−0.383; 1.000]	1.000	P	0.000	[1.000; 1.000]	1.000	P	0.000	[1.000; 1.000]	0.660	G	0.357	[−0.477; 1.000]
**Test item 4** Catching an object under water	1.000	P	0.000	[1.000; 1.000]	1.000	P	0.000	[1.000; 1.000]	1.000	P	0.000	[1.000; 1.000]	1.000	P	0.000	[1.000; 1.000]	1.000	P	0.000	[1.000; 1.000]
**Test item 5** Floating on the back (i.e., back star	1.000	P	0.000	[1.000; 1.000]	0.719	G	0.310	[−0.268; 1.000]	1.000	P	0.000	[1.000; 1.000]	0.673	G	0.332	[−0.383; 1.000]	1.000	P	0.000	[1.000; 1.000]
**Test item 6** Floating on the front (i.e., front star)	1.000	P	0.000	[1.000; 1.000]	1.000	P	0.000	[1.000; 1.000]	0.660	G	0.357	[−0.477; 1.000]	1.000	P	0.000	[1.000; 1.000]	1.000	P	0.000	[1.000; 1.000]
**Test item 7** Water entry by slide	1.000	P	0.000	[1.000; 1.000]	1.000	P	0.000	[1.000; 1.000]	1.000	P	0.000	[1.000; 1.000]	0.719	G	0.310	[−0.268; 1.000]	1.000	P	0.000	[1.000; 1.000]
**Test item 8** Pushing from the wall and gliding under water	1.000	P	0.000	[1.000; 1.000]	1.000	P	0.000	[1.000; 1.000]	0.719	G	0.310	[−0.268; 1.000]	1.000	P	0.000	[1.000; 1.000]	1.000	P	0.000	[1.000; 1.000]
**Test item 9** Leg propulsion on the back	0.628	G	0.367	[−0.539; 1.000]	1.000	P	0.000	[1.000; 1.000]	1.000	P	0.000	[1.000; 1.000]	1.000	P	0.000	[1.000; 1.000]	0.628	G	0.367	[−0.539; 1.000]
**Test item 10** Leg propulsion on the front	0.644	G	0.382	[−0.572; 1.000]	1.000	P	0.000	[1.000; 1.000]	1.000	P	0.000	[1.000; 1.000]	0.660	G	0.357	[−0.477; 1.000]	1.000	P	0.000	[1.000; 1.000]
**Test item 11** Water entry by jumping	1.000	P	0.000	[1.000; 1.000]	1.000	P	0.000	[1.000; 1.000]	1.000	P	0.000	[1.000; 1.000]	1.000	P	0.000	[1.000; 1.000]	1.000	P	0.000	[1.000; 1.000]
**Test item 12** Water entry by diving	1.000	P	0.000	[1.000; 1.000]	1.000	P	0.000	[1.000; 1.000]	1.000	P	0.000	[1.000; 1.000]	1.000	P	0.000	[1.000; 1.000]	0.628	G	0.367	[−0.539; 1.000]
**Test item 13** Water exit by climbing out	1.000	P	0.000	[1.000; 1.000]	1.000	P	0.000	[1.000; 1.000]	0.673	G	0.332	[−0.383; 1.000]	1.000	P	0.000	[1.000; 1.000]	0.719	G	0.310	[−0.268; 1.000]
**Test item 14** Vertically treading water	1.000	P	0.000	[1.000; 1.000]	1.000	P	0.000	[1.000; 1.000]	1.000	P	0.000	[1.000; 1.000]	1.000	P	0.000	[1.000; 1.000]	1.000	P	0.000	[1.000; 1.000]
**Test item 15** Longitudinal axis rotation	1.000	P	0.000	[1.000; 1.000]	1.000	P	0.000	[1.000; 1.000]	1.000	P	0.000	[1.000; 1.000]	1.000	P	0.000	[1.000; 1.000]	1.000	P	0.000	[1.000; 1.000]
**Test item 16** Transverse axis rotation	1.000	P	0.000	[1.000; 1.000]	1.000	P	0.000	[1.000; 1.000]	1.000	P	0.000	[1.000; 1.000]	1.000	P	0.000	[1.000; 1.000]	0.719	G	0.310	[−0.268; 1.000]
**Test item 17** Sagittal axis rotation	1.000	P	0.000	[1.000; 1.000]	1.000	P	0.000	[1.000; 1.000]	1.000	P	0.000	[1.000; 1.000]	1.000	P	0.000	[1.000; 1.000]	1.000	P	0.000	[1.000; 1.000]

AC2 = Gwet’s Agreement Coefficient 2; LoA = Level of Agreement; M = moderate (0.41–0.60); G = good (0.61–0.80); VG = very good (0.81–0.99); P = perfect (1.00); SE = standard error; CI = confidence interval.

**Table 7:** Test re-test agreement per rater.

Test Component	Rater A	Rater B	Rater C	Rater D	Rater E
Test video 1	88.24%	100%	94.12%	88.24%	88.24%
Test video 2	100%	100%	88.24%	94.12%	88.24%
Test video 3	100%	100%	100%	100%	94.12%
Test video 4	100%	88.24%	100%	100%	94.12%
**Test item 1** Move forward as a crocodile	100%	100%	100%	100%	75%
**Test item 2** Standing and submersion	100%	100%	100%	100%	100%
**Test item 3** Blowing bubbles under water	100%	75%	100%	100%	75%
**Test item 4** Catching an object under water	100%	100%	100%	100%	100%
**Test item 5** Floating on the back (i.e., back star)	100%	75%	100%	75%	100%
**Test item 6** Floating on the front (i.e., front star)	100%	100%	75%	100%	100%
**Test item 7** Water entry by slide	100%	100%	100%	75%	100%
**Test item 8** Pushing from the wall and gliding under water	100%	100%	75%	100%	100%
**Test item 9** Leg propulsion on the back	75%	100%	100%	100%	75%
**Test item 10** Leg propulsion on the front	75%	100%	100%	75%	100%
**Test item 11** Water entry by jumping	100%	100%	100%	100%	100%
**Test item 12** Water entry by diving	100%	100%	100%	100%	75%
**Test item 13** Water exit by climbing out	100%	100%	75%	100%	75%
**Test item 14** Vertically treading water	100%	100%	100%	100%	100%
**Test item 15** Longitudinal axis rotation	100%	100%	100%	100%	100%
**Test item 16** Transverse axis rotation	100%	100%	100%	100%	75%
**Test item 17** Sagittal axis rotation	100%	100%	100%	100%	100%

## Data Availability

The data presented in this study are available on request from the corresponding author.
